# The Older, the More Forgiving? Characteristics of Forgiveness of Chinese Older Adults

**DOI:** 10.3389/fpsyg.2021.732863

**Published:** 2021-12-06

**Authors:** Linjin Tao, Tingting Zhu, Yanglu Min, Mingxia Ji

**Affiliations:** ^1^School of Psychology, Nanjing Normal University, Nanjing, China; ^2^Institute of Medical Humanities, Nanjing Medical University, Nanjing, China; ^3^Wuxi Qingshan Senior High School, Wuxi, China; ^4^Guangming Branch of Shenzhen Institute of Education Sciences, Shenzhen, China

**Keywords:** forgiveness, older adults, implicit association test (IAT), Chinese, Gender difference, explicit forgiveness, implicit forgiveness

## Abstract

This study explores the characteristics of forgiveness in the aging cohorts, which is regarded to be associated with healthy outcomes. Data were drawn from a sample of 308 older adults (aged from 60 to 98 years) who completed the forgiveness questionnaire: forgiving others of The Heartland Forgiveness Scale (HFS) to examine explicit forgiveness, and among the participants, 44 older adults were administrated on the variant single category of implicit association test (SC-IAT) to examine the implicit forgiveness. The results revealed that (1) there is no correlation between explicit forgiveness and implicit forgiveness of older adults. (2) The result of explicit forgiveness is relatively high while that of implicit forgiveness is relatively low. (3) There was no significant correlation between explicit forgiveness and age, but there was significant difference between age groups, as forgiveness tendency of the elderly had a trough in the age group of 70–79 and then rebounded. (4) Implicit forgiveness was significantly correlated with age, and the difference between age groups was marginal. The forgiveness tendency of the elderly over 80 years old was significantly higher than that of the other two age groups. (5) Gender differences are found in both explicit and implicit forgiveness. The findings indicated that (1) explicit and implicit measures in this study have assessed independent and complementary aspects of forgiveness tendency in older adults. (2) Implicit forgiveness falls behind explicit forgiveness, and true internal forgiveness is difficult and rare in older adults according to data analysis. (3) The trend of explicit forgiveness with age is not obvious, because explicit forgiveness in the middle old age group presents an inflection point. However, implicit forgiveness increases slowly with age. (4) Women excel men in scores obtained with both explicit and implicit measures for forgiveness.

## Introduction

There is a well-known ancient Chinese poem “life has been rare for 70 years,” in the past, 70 years was very old age. However, with the progress of science and technology and the promotion of the active aging process, people with long life become less rare. Erikson’s stage theory of psychological development suggested that human development has eight stages in which an individual faces specific conflicts, and successful resolution of each conflict leads to a stronger sense of identity and more rewarding interactions with people. As people grow old and come to the final stage of maturation, they feel the need to complete life in a satisfactory way by gaining closure and endowing their life experiences with meaning, thus achieving ego integrity, which calls for “the acceptance of one’s one and only life cycle” (Erikson, 1994; Hantman and Cohen, 2010). In addition, with no doubt, this process requires compromise, tolerance, and acceptance (Kaleta and Mróz, 2018). So the act of forgiveness will probably have a crucial effect on meaning in later life since one of the major sources of meaning identified in the literature is interpersonal relationships (Hantman and Cohen, 2010). When looking back on the past, it is also a common thing for elderly people to recall interpersonal conflicts in the past. They may resent or give new meaning to some unfair treatment they have experienced. How do they end these grievances in their hearts? According to the socioemotional selectivity theory of Carstensen, perceiving the limited time horizon is important for selecting goals, activities, and preferences (Carstensen et al., 1999). When people with the growth of age realize that their time is limited rather than unrestrained, the emotional experience begins to prevail over knowledge acquisition. For example, young people perceive that the future time is abundant and give priority to acquiring knowledge; while the elderly people tend to choose emotion regulation as a goal. That is to say, old people may choose to complete life in a satisfactory way as Erikson said through regulating inner emotions instead of pursuing who is right or who is wrong. Strategies might be required for feeling satisfied with life. Older adults would engage in strategies that minimize negative social experiences and maximize positive ones by avoiding interpersonal conflicts (Allemand and Olaru, 2021). Since findings to date have shown that forgiveness has the power of restoring difficult relations and improving the wellbeing of individuals, forgiveness is thought to be one strategy for maximizing positive emotions and a possible coping style to interpersonal transgression (Worthington, 2005; Wilkowski, 2010).

Forgiveness is frequently conceptualized in terms of decreased negative affect (e.g., bitterness, anger, and hostility), negative cognitions (e.g., thoughts of revenge), negative motivation (e.g., tendency to avoid any contact with the offender), and negative behavior (e.g., verbal aggression) toward the offender (Enright et al., 1989; Mccullough et al., 1998; Rye and Pargament, 2002; Kaleta and Mróz, 2018). It is also commonly defined as the ability of an individual to forgive across a variety of situations and relationships, which can be viewed as a personal disposition (McCullough, 2000; Berry et al., 2001). The dispositional forgiveness is also called trait forgiveness, or forgivingness corresponding to the situational forgiveness mentioned above. Some scholars believed that the disposition to forgive others is more robustly associated with the quality of life than a single act of forgiveness (Kaleta and Mróz, 2018). Scholars have also established different objects of forgiveness as follows: forgiveness of others, forgiveness of self, forgiveness of situations beyond the control of anyone, and forgiveness by God. Nevertheless, in this study, we only focused on the forgiveness of others, that is, interpersonal forgiveness.

Past studies have shown that forgiveness among older adults may signal “a life well lived” as those who forgive are more likely to report healthy behaviors and have more social support (Lawlerrow and Piferi, 2006), and forgiveness is also positively associated with life satisfaction among older adults (Toussaint et al., 2001; Krause and Ellison, 2003; Ermer and Proulx, 2016). Besides, the level of forgiveness is positively associated with the wellbeing of individuals (Hill and Allemand, 2011; Lee et al., 2012), interpersonal relationship (Fingerman and Charles, 2010), and physical and psychological health (Toussaint et al., 2001; Ermer and Proulx, 2016) of the older adults. Similar to the evolution of social emotions and cognitive skills, the ability to forgive is also developmental (Allemand and Steiner, 2011). Several studies reported that older adults are more willing to forgive others than younger adults, with richer life experiences and skills in responding interpersonal transgression (Ghaemmaghami et al., 2011; Steiner et al., 2011). However, there is a lack of research to subdivide the “aging” population and focus on the details of forgiveness among this group. Since forgiveness has an obvious effect on health, is it true that the older they are, the more inclined they are to forgive? Do old people really have a forgiving disposition?

Forgiveness has often been assessed *via* self-report, which is often criticized for being liable to social desirability. It is difficult to capture the subconsciousness of subjects by self-report. As a result, one might explicitly state that one has forgiven an offender, yet implicitly continue harboring a grudge (Fincham, 2009). To remedy these limitations, the forgiveness Implicit Association Test (IAT) is developed (Greenwald et al., 2003; Zhu, 2012; Fatfouta et al., 2014). In the sample of 104 students (70 women; *M* age = 25.10 years, SD = 4.39), Fatfouta et al. has revealed that the forgiveness IAT was highly reliable, moderately stable over time, demonstrated incremental validity, and this research could advance personality research beyond what is known from self-report measures (Fatfouta et al., 2014). Zhu (2012) had found similar results in Chinese Middle School samples. The IAT effect, defined as response-latency difference between congruent and incongruent pairings, thus reflects associative links between the self and a trait concept (in our case, forgiveness) and may be referred to as implicit self-concept of personality (Schnabel and Asendorpf, 2010). The term “implicit” reflects the idea that preference decisions of individuals operate outside of conscious awareness. As such, the IAT has the potential to address shortcomings of self-report, particularly in relation to forgiveness, what is more, differences between implicit and explicit processes that do not arise simply because the two are measured differently, but because they are distinct constructs (Goldring and Strelan, 2017).

To date, however, few studies have attempted to look at the implicit self-concept of forgiveness among older adults. It is important to understand the propensity to forgive others in older adults as well as to examine whether there are quantitative and qualitative changes in their forgiveness. This study sought to investigate the disposition to forgive others and the possible age or gender differences in older adults, with both explicit and implicit measures, so as to provide evidence of intervention for improving the level of forgiveness, life satisfaction, and subjective wellbeing in older adults.

To sum up, our hypotheses are as follows: old people have a forgiving disposition *via* explicit and implicit measures (Hypothesis 1); the forgiveness tendency of the elderly people increases with age *via* explicit and implicit measures (Hypothesis 2); women excel men in scores obtained with both explicit and implicit measures for forgiveness (Hypothesis 3).

## Materials and Methods

### Participants

Using the method of stratified sampling and convenient sampling, 391 older adults were selected from 7 communities and 1 elderly care institution in Nanjing, among which 308 valid questionnaires were recalled (155 female, 142 male; mean age = 71.69 years, SD = 7.50 years, ranging from 60 to 98 years; 11 of them did not report their gender in the questionnaires). Questionnaires completed by 83 participants were not included in the analyses because they did not meet study criteria, such as provided incomplete responses or provided responses that were not valid. Consequently, a total of 308 participants were included in the analyses. Since this study allows withdrawal, the recovery rate of the questionnaire is not very ideal.

Besides the explicit forgiveness test for all participants, we randomly assigned 50 of them to be administrated on a single category of IAT (SC-IAT) to examine the implicit forgiveness. Finally, the data of 44 participants were considered valid (21 female, 23 male; mean age = 69.09 years, SD = 8.27 years, ranging from 61 to 98 years). It is because a few of them incapable of performing on the computer and the data with response time over 3,000 ms or error rate higher than 20% were eliminated.

Participants were told that participation was completely voluntary, and they were free to withdraw whenever they wanted. After obtaining informed consent from participants, paper-and-pencil questionnaires were administered in the community activity center. For those who had literate and visual problems, they were assisted by trained student assistants from the research team during the survey process.

Participants signed informed consent forms and could withdraw from this study at any time. The confidentiality of the participants is guaranteed in several ways, that is, the names of all participants are anonymous.

### Instruments

#### Explicit Measure

This study applies Heartland Forgiveness Scale (HFS) (Thompson et al., 2005). HFS contains 18 items for three subscales with 6 items as follows: forgiveness of self, others, and situations. It is a seven-point Likert-type scale ranging from “almost always false than true” (1) to “almost always true of me” (7). In our study, we employed the subscale of forgiving others, with items such as “If others mistreat me, I continue to think badly of them.” Internal consistency alpha was found to be 0.67.

#### Implicit Measure

In this study, we used the variant of the IAT, SC-IAT, which was also called the Wigboldus IAT (WIAT) paradigm developed by Karpinski and Hilton (2001). This study programs and presents the stimuli in Inquisit 4. The stimulus word sets for attribute categories (forgiveness or “Kuan Shu” in Chinese: Kuan Shu De, Kuan Rong De, Yuan Liang De, Ti Liang De, He Jie De, Li Jie De, Rao Shu De, Tong Qing De; unforgiveness or “Bu Kuan Shu”: Fen Nu De, Zeng Wu De, Yuan Hen De, Bao Fu De, Di Dui De, Zhi Ze De, Tao Bi De, and Hui Bi De) stemmed from the study by Zhu in which the author filtered the above two sets of eight Chinese words, respectively. The concept words of “self” included the following eight Chinese words: Zi Ji, Zi Wo, Wo Men, Wo De, Zan Men, Zi Ge, Zi Jia, and Zi Ji De (Zhu, 2012).

#### Single Category of Implicit Association Test Procedure

There are four blocks (Table 1). In training Block 1, category words “self” and “forgiveness” are presented on the up-left side of the screen while “unforgiveness” is on the upright. The stimulus words belonging to categories of “forgiveness”/“unforgiveness,” or “self” appear in the middle of the screen successively. Participants are required to press “E” if the stimulus word belongs to the up-left category and press “I” if the stimulus word belongs to the upright category. Each stimulus word belongs to one category only. If a stimulus word is categorized under the wrong category, an “X” appears on the screen, and the categorization of the stimulus word has to be rectified by pressing the correct key. Participants are asked to respond quickly and accurately. Block 2 is a formal test, which resembles Block 1. Block 3 is also a practice procedure similar to Block 1 with the category “forgiveness” on the up-left while “self” and “unforgiveness” on the upright. Block 4 is a formal test procedure of Block 3.

**TABLE 1:** Sequence of blocks in the forgiveness/unforgiveness single category of implicit association test (SC-IAT).

Block	No. of trials	Function	“E”key assignment	“I” key assignment
1	20	Practice	Self + forgiveness	Unforgiveness
2	40	Test	Self + forgiveness	Unforgiveness
3	20	Practice	Forgiveness	Self + Unforgiveness
4	40	Test	Forgiveness	Self + Unforgiveness

*To counterbalance the order effect, the above sequence was applied to the odd number of participants, while the sequence for the even number of participants was Block 3 – Block 4 – Block 1 – Block 2.*

We calculated *D* scores following the scoring algorithm outlined by Greenwald et al. (2003) The greater the *D* score is, the larger the differences are in reaction preference toward the pairings of two opposite sets of stimulus words. That is to say, higher scores reflect stronger automatic associations between me and forgiving (vs. me and vengeful), and therefore, a more forgiving implicit self-concept. The data process followed the following procedure: first, calculate SD (*Dp* and *Dt*), respectively, for two practice blocks (i.e., Block 1 and Block 3) and two test blocks (i.e., Block 2 and Block 4); second, calculate mean score of reaction time for four Blocks (i.e., *M*_1_, *M*_2_, *M*_3_, and *M*_4_); third, obtain *D1* by dividing the reaction time discrepancy between Block 1 and Block 3 by the SD of practice blocks, and in the same way, obtain *D2* for Block 2 and Block 4; finally, average *D1* and *D2* to get *D*. The formulas are as follows:


(1) $$D=\frac{D\,1+D\,2}{2}\,\ldots \,\ldots$$

(2) $$D\,1=\frac{M_{3}-M_{1}}{D_{p}}\,\ldots \,\ldots$$

(3) $$D\,2=\frac{M_{4}-M_{2}}{D_{t}}\,\ldots \,\ldots$$

## Results

### Explicit Forgiveness

Our data analysis has found that the mean score of explicit forgiveness on HFS is 4.63 (SD = 0.80) for this sample (Table 2), between “not sure” and “moderately true of me,” which indicates that there is a tendency to forgive.

**TABLE 2:** Age differences of explicit and implicit forgiveness.

		All^0^		60 to 69 years old ^1^		70 to 79 years old ^2^		above 80 years old ^3^		*F*	*p*
							
		M	SD	M	SD	M	SD	M	SD		
Explicit measure	Forgiving others of HFS	4.63	0.80	4.68	0.81	4.48	0.75	4.84	0.81	4.30*	0.014
											2 < 1
											2 < 3
	Forgiving others of HFS – positive forgiveness	4.40	1.22	4.54	1.20	4.21	1.19	4.51	1.31	2.51^Δ^	0.083
											2 < 1
	Forgiving others of HFS – reduction of unforgiveness	4.85	1.20	4.83	1.23	4.74	1.18	5.17	1.11	2.26	0.106
											1 < 3^Δ^
											2 < 3
Implicit measure		0.10	0.61	0.07	0.57	−0.08	0.57	0.61	0.67	2.84^Δ^	0.07
											1 < 3
											2 < 3

*^0^N_1_ = 307 (explicit forgiveness), N_2_ = 44 (implicit forgiveness).*

*^1^n_1_ = 136 (explicit forgiveness of the young age group from 60 to 69 years), n_2_ = 27 (implicit forgiveness of the young age group from 60 to 69 years).*

*^2^n_1_ = 122 (explicit forgiveness of the young age group from 70 to 79 years), n_2_ = 11 (implicit forgiveness of the young age group from 70 to 79 years).*

*^3^n_1_ = 49 (explicit forgiveness of the young age group above 80 years), n_2_ = 6 (implicit forgiveness of the young age group above 80 years).*

^Δ^P < 0.1, *P < 0.05, same below.

#### Age Differences

Correlation analysis showed that there was no significant correlation between explicit forgiveness and age in this sample. *r*_(HFS)_ = 0.009 and *p* = 0.877 > 0.05. According to the categorization of WHO in GBD2000, the participants were divided into the following three groups: the young old (60–74 years), the old old (75–89 years), and the very old (above 90 years). ANOVA was carried out to compare HFS scores across the four age groups. The mean scores of the three groups on HFS were 4.60, 4.64, and 5.42, with a tendency to increase; however, no significant group differences were found according to variance analysis (*F*_(2,304)_ = 2.081 and *p* > 0.05). However, when the participants were divided into the other three groups as follows: young old age group (60–69 years), middle old age group (70–79 years), and old old age group (above 80 years), things are different. The results of ANOVA are shown in Table 2. The mean scores of three groups are 4.68 (SD = 0.81), 4.48 (SD = 0.75), and 4.84 (SD = 0.81), and a significant main effect of age was found for general forgiveness (*F*_(2,304)_ = 4.30; *p* = 0.014 < 0.05) (Table 2). The *post hoc* test showed that the middle old age group (70–79 years) participants reported a significantly lower level of forgiveness than the young old age group (60–69 years) (*p* = 0.038) and old old age group (above 80 years) (*p* = 0.007). As for the forgiving others of HFS – positive forgiveness dimension, there was a marginal difference between the three groups (*F*_(2,304)_ = 2.514; *p* = 0.083). The *post hoc* tests showed that the mean score of the middle old age group (70–79 years) was significantly lower than the mean score of the young old age group (60–69 years) (*p* = 0.033). No significant main effect of age was found for reducing the unforgiveness of others (forgiving others of HFS – reduction of unforgiveness). However, the *post hoc* tests showed that the mean score of old old age group (above 80 years) was significantly higher than that of the middle old age group (70–79 years) (*p* = 0.036) and was marginally higher than that of the young old age group (60–69 years) (*p* = 0.087).

#### Gender Differences

As shown in Table 3, the mean scores of male and female participants on HFS were 4.48 (SD = 0.72) and 4.78 (SD = 0.84), respectively. Statistically significant gender differences were found according to *T*-test analysis indicating a higher tendency of forgiveness for women than men in this study. Similarly, a significant main effect of gender was found for overcoming unforgiveness. No significant gender difference was found for positive forgiveness.

**TABLE 3:** Gender differences of explicit and implicit forgiveness.

	Explicit forgiveness			Implicit forgiveness
	Forgiving others of HFS	Forgiving others of HFS – positive forgiveness	Forgiving others of HFS – reduction of unforgiveness	
Male (142)	4.48 ± 0.72	4.40 ± 1.09	4.55 ± 1.16	
Female (155)	4.78 ± 0.84	4.44 ± 1.31	5.10 ± 1.18	
*t*	−3.25**	−2.25	−4.06***	
Total (*N* = 308)	4.63 ± 0.80	4.40 ± 1.22	4.85 ± 1.20	
male (23)				−0.08 ± 0.61
female (21)				0.31 ± 0.56
*t*				−2.20*
Total (*N* = 44)				0.10 ± 0.61

*p < 0.05; **p < 0.01; ***p < 0.001.

### Implicit Forgiveness

The results of data analysis for implicit forgiveness tests in this study are shown in Tables 2, 3 (*M* = 0.10 and SD = 0.61). A score below 0 means that a certain associative strength exists between self and unforgiveness; meanwhile, a score above 0 indicates a certain associative strength between self and forgiveness. With a standard of 0, the *T*-test analysis with the mean score of implicit forgiveness found that there is no difference in associative strength for self/forgiveness and self/unforgiveness (*t* = 1.072 and *p* = 0.29 > 0.05).

#### Age Differences

A significant correlation has been found with analysis for scores on implicit forgiveness and age (*r*_(SC–IAT)_ = 0.319 and *p* = 0.035 < 0.05). The result of variance analysis for implicit forgiveness scores among the three age groups is also shown in Table 2, and the mean scores were 0.07 (SD = 0.57), −0.08 (SD = 0.57), and 0.61 (SD = 0.67). Marginal significant differences among the three groups were found (*F*_(2,41)_ = 2.837, *p* = 0.070), and the *post hoc* tests showed that the mean score of the old old age group (above 80 years) was significantly higher than that of the middle old age group (70–79 years) (*p* = 0.025) and that of the young old age group (60–69 years) (*p* = 0.046), which means older participants, more likely to perceive themselves as forgiving, associated self and forgiveness more than the young old participants did.

#### Gender Differences

As shown in Table 3, the mean scores for implicit forgiveness were −0.08 (SD = 0.61) for men and 0.31 (SD = 0.56) for women, one being below zero and the other above. According to the result of *T*-test, significant gender differences were found, meaning female older adults had scored significantly higher in the implicit forgiveness test than male older adults did in this sample. The male older adults showed zero associative strength for self/forgiveness correlation, and there was even a tendency for unforgiveness; however, the female older adults had demonstrated low associative strength for self/forgiveness. That is, the female older adults are more likely to consider themselves as being forgiving than the male older adults are.

#### Explicit-Implicit Correlation

As shown in Table 4, scores on forgiving others of HFS were significantly correlated, while no significant correlation had been found for scores between implicit measure and forgiving others of HFS. This result indicates the existence of different aspects of forgiveness (implicit vs. explicit) and the two are independent of each other.

**TABLE 4:** Correlation of explicit and implicit forgiveness in older adults.

	Forgiving others of HFS	Forgiving others of HFS – positive forgiveness	Forgiving others of HFS – reduction of unforgiveness	Implicit forgiveness
Forgiving others of HFS	1			
Forgiving others of HFS – positive forgiveness	0.668**	1		
Forgiving others of HFS – reduction of unforgiveness	0.650**	−0.131*	1	
Implicit forgiveness	−0.100	−0.080	−0.058	1

*p < 0.05; **p < 0.01.

## Discussion

This study has tested the forgiveness tendency in older adults with explicit and implicit measures and confirmed these two independent and complementary aspects of forgiveness, which is consistent with previous studies (Allemand and Steiner, 2011; Bast and Barnes-Holmes, 2014; Fatfouta et al., 2014). Nosek had suggested that usually no significant correlation was found for the data obtained from explicit and implicit measures of a psychological construct (Nosek, 2005). Fatfouta et al. (2014) suggested that IAT of forgiveness could advance personality research beyond what is known from self-report measures.

Mean scores for explicit forgiveness from forgiving others of HFS in this study were close to “moderately true of me” rather than “not sure,” consistent with results for explicit forgiveness of the young old mentioned in the study by Allemand and Steiner (2011). In contrast, the mean score for implicit forgiveness was 0.10, between “unforgiving” and “forgiving,” which could be seen as “not sure.” Therefore, the degree of implicit forgiveness seems to be lower than that of explicit forgiveness. In another word, the implicit forgiveness fell behind in the explicit forgiveness of older adults. Thus, Hypothesis 1 – old people have a forgiving disposition *via* explicit and implicit measures – is partly supported.

Previous studies have indicated that older adults are, on average, more willing to forgive others than younger adults (Steiner et al., 2011). There is also an intervention study demonstrating that the older adults are being more willing to forgive (Allemand et al., 2013). According to this presumption, the forgiveness scores of older participants would be higher; however, the correlation of age and explicit forgiveness has not been found in this study as data analysis did not reveal differences for the young old, the old old, and the very old. This may be interpreted as the development of forgiveness over the age of 60 years is relatively stable. Allemand and Flückiger (2020) selected 73 older adults (the sample consisted of predominately healthy young-old adults (65–74 years)) and randomized them to two intervention conditions that helped them to either understand the transgression in a more adaptive way or to practice new behaviors and skills to manage the transgression. The findings indicated that both conditions (i.e., a learning-oriented group and an action-oriented group) resulted in decreases in revenge, transgression-related thoughts and feelings, negative affect, and psychological distress. However, their study also demonstrated that avoidance and learning-oriented revenge response first decreased and then recovered and generally returned to the pretest time though learning-oriented revenge response declined and benevolence response rise. As for negative emotion, it always decreased before the post-test, but then rebounded, indicating that although the intervention is effective in reducing negative emotion, it also shows that change is quite difficult. When the intervention is withdrawn, it will rebound. These processes often cannot be initiated by oneself but must be specifically activated through intervention. The results are similar to those of our study.

Correlation analysis showed that there was no correlation between age and explicit forgiveness. However, when being divided into age groups, the results of ANOVA showed that the explicit forgiveness of the elderly people in the age group of 70–79 years was significantly lower than that in the other two age groups, indicating that the self-reported forgiveness tendency of the elderly people had a trough in the age group of 70–79 years and then rebounded; from the two dimensions of the forgiveness questionnaire, the positive dimension of explicit forgiveness of the elderly people has a significant downward trend in the age group of 70–79, while in the negative dimension (i.e., the tendency to overcome unforgiveness), the tendency of the elderly people over 80 years to overcome unforgiveness is significantly higher than that of the elderly people over 70–79 years and slightly higher than that of the elderly people under 70 years (marginal significant), that is, the trend that explicit forgiveness increases with age is more reflected in overcoming unforgiveness. This trend is consistent with that of implicit forgiveness. After being divided into age groups, the results of ANOVA showed that the implicit forgiveness of the elderly people over 80 years was significantly higher than that of the first two age groups. Hypothesis 2 – the forgiveness tendency of the elderly people increases with age *via* explicit and implicit measures – is also partly supported.

These results are consistent with the previous research (Kaleta and Mróz, 2018). They argued that as people age, they do not show a specific tendency to become more benevolent toward their transgressors, although they do tend to reduce their hostile emotions, thoughts, and behaviors toward offenders in a variety of situations. Similarly, Allemand and Olaru (2021) found a substantial decrease in the tendency to show avoidance from adult to old people.

In contrast, as for implicit forgiveness, which is more close to subconsciousness, a certain correlation of age and forgiveness has been found (*R* (SC-IAT) = 0.319 and *p* = 0.035 < 0.5). This means that the strong correlation for self and forgiveness increases with age. The older adults consider themselves to be more forgiving than the younger ones. Implicit forgiveness is considered to be close to personal traits and predictive of actual negative responses during interpersonal transgression (Fatfouta et al., 2014). Previous studies showed that transgression frequency and intensity partially explained age differences in forgivingness (Steiner et al., 2011). Hill et al. had also pointed out that the experiences gained through interpersonal transgression would increase the motive to forgive others (Hill and Allemand, 2011). All these studies had assessed explicit forgiveness through oral reports, assuming that individual forgiveness could only be understood in specific interpersonal conflicts. However, implicit measures of forgiveness, being cross-situational, have captured more lively and authentic trait of forgiveness among the older adults under non-specific situations in an easier way.

As proposed by Park and Enright (1997), the developmental sequence in understanding forgiveness is related to age. There are three types of forgiveness as follows: “Revenge Forgiveness,” “Pseudo Forgiveness,” and “Internal Forgiveness.” The most developed one, “Internal Forgiveness,” is unconditional and authentic as a person follows the inner principles of kindness and love so as to forgive others. Meanwhile, “Pseudo Forgiveness” means that the individual appears to be forgiving while still feeling frustrated and depressed (Park and Enright, 1997). Park et al. suggested that few people have developed “Internal Forgiveness.” This present study supports this statement with evidence that it is difficult for the individual to develop authentic forgiveness. Though at the level of overcoming unforgiving, it shows that the older they are, the more they can overcome unforgiving. However, on the external performance, it shows that the elderly people aged 70–79 years are more reluctant to show a positive aspect of forgiveness, that is, they are unwilling to show benevolence to the offender. Thus, as people age, they do not show a specific tendency to become more benevolent toward their transgressors, although they do tend to reduce their hostile emotions, thoughts, and behaviors toward offenders in a variety of situations. These results are consistent with the previous research on episodic forgiveness which revealed revenge and avoidance motivation to be related to age, whereas benevolence motivation is not associated with age (Kaleta and Mróz, 2018). One has more complex motives to show benevolence.

According to Ghaemmaghami et al. (2011), compared with middle-aged and young people, older people report more offended experiences as dismissal, forced to leave home, being rejected, being treated unfairly, unjustly, and being harassed/experiencing unjust distribution of money. As for the old adults aged 70–79 years in the Chinese context, they are less needed than those aged 60–70 years, probably experiencing more above offends than other old groups. The elderly people aged 60–69 years in Chinese families retire from their jobs to help their children raise the third generation, which can make them feel needed. The needs of the elderly people aged 70–80 years may be reduced, while the elderly people over 80 years turn to focus on themselves and live longer than others with the reduction of their personal energy. This may enhance their self-esteem, so it is easier to forgive the unfairness of the past.

On the whole, to put the results of explicit and implicit forgiveness found in this study together, the developmental characteristics of older adults can be summarized as follows: scores on explicit forgiveness are relatively higher but showed fluctuations; scores on implicit forgiveness are lower in general with certain age differences from the young old to the very old.

Why does such inconsistency with age differences exist among explicit and implicit forgiveness? Perhaps, it could be explained by the specific Chinese family structure and social culture. In China, the young old usually still maintain the previous social relationship after the recent retirement, meanwhile devoting themselves to taking care of the grandchildren in the family. Therefore, interpersonal interaction is still an important aspect of their life. Chinese culture always holds the principle of “return good for good, justice in return for injustice” in interpersonal relationships (Confucius, 2016), while the moral requirement of “being benevolent” (Mencius, 1935) and “repaying injury with kindness” (Lao, 1929) has exerted a subtle influence on the individuals as well. In China, we used phrases to describe the older adults positively and negatively such as “be of noble character and high prestige” and “not worthy of respect.” The traditional Chinese society values older adults as the model of their family and community. Therefore, higher moral expectations are put on the older adults than the young. For the young old who are still in complex interpersonal relationships, personal favors and saving face are important factors influencing how they deal with social interactions. This is in accordance with the study by Fu (2006).

Therefore, the scales, liable to the inevitable social expectation effect, had examined the “ideal self” of the older adults (especially the young old) which was “I should be forgiving,” while the implicit association test, more relative to the true responses of subconsciousness, had captured to some degree their “real self,” which was “I cannot forgive.” Moreover, for the very old, interpersonal relationship is no longer their primary concern. Instead, they confront the inner world with issues such as fulfillment and loss, while those unfinished businesses are easily dealt with internally. According to the socioemotional selectivity theory (Carstensen, 2006), since older adults increasingly recognize that they have limited time left to live, their goals related to deriving emotional meaning from life are prioritized over goals that maximize long-term payoffs (e.g., acquisition of knowledge). To maintain emotional wellbeing, older adults will be more willing to select internal forgiveness with age. Meanwhile, with the physical deterioration, they are more often taken care of, and past interpersonal traumas are understood with new insights in confronting the death topic. Therefore, as the age increases among the older adults, the explicit and implicit forgiveness become more consistent and present with a higher level.

This study has also provided evidence of gender differences in both explicit and implicit forgiveness as women scored higher than men in this sample. Hypothesis 3 – women excel men in scores obtained with both explicit and implicit measures for forgiveness – is totally supported. It is consistent with the meta-analysis of forgiveness of Miller et al. (2008), which reported that women are more forgiving than men are, and that difference value is between low and moderate. The explanation, according to the theory of moral development of Gilligan, might be that men demonstrate “justice-centered” morals while women demonstrate “relationship-centered” morals (Gilligan, 1977). In this case, only the elimination of unfairness could induce forgiveness in men as they require justice; otherwise, unconditional forgiveness would rarely happen. Meanwhile, women consider how to maintain the relationship by taking the perspective of others so that their warmth and caring could reduce hostility caused by objective unfairness, and it is easier for them to show forgiveness.

It is worth noting that, in the literature review of previous studies of forgiveness, the results for gender difference could be affected by the factors of measures, participants, forgiveness categories, cultural influence, and so on. For example, no gender differences were found in the study by Zhu (2012) of adolescence forgiveness with implicit association test. Therefore, further studies are needed for the study of gender differences in the forgiveness of older adults.

The above discussion implicates that, in practical work with older adults, we should not neglect the effect of interpersonal transgression, especially for those in middle old age, despite their appearance of forgiving. The caring of their mental health should be focused on the development of internal forgiveness in the perspective of actual interpersonal conflicts so that they would maintain mental harmony as their inner world coincides with external appearance.

This study has confirmed that studies of implicit forgiveness verify and complement those of the explicit forgiveness. The parallel studies enable us to fully grasp the relationship between forgiveness and age as well as forgiveness and gender. In future studies, researchers may expand the range of age and make a further comparison for age differences with both measures of forgiveness.

## Limitation

The number of the three age groups varies widely. It is difficult to find the middle old age group and old old age group. However, it was a valuable attempt to look at the implicit forgiveness among the older adults rather than comparing the difference between younger and older adults. Future research should include a broader range of older adults to explore the developmental trajectories of forgiveness. The assessments used in this study required that the participants should be literate, which limited the representativeness of this sample, while the present literature review has not mentioned the influence of educational attainments on forgiveness (Yali and Ye, 2008). Moreover, it requires approximately 45 min to 1 h to finish all the tests in this study, and the implicit test was operated on the computer. For a large part of the participants, they were tired after the questionnaires and gave up the implicit test due to physical condition. This resulted in the fact that the sample of explicit and implicit forgiveness was inconsistent, and the data of this sample was not so perfect. In contrast, more than 40 older adults participated in the implicit test, and with the diffused age structure of this sample, we can still rely on the data analysis to explain implicit forgiveness in older adults.

## Conclusion

The conclusions of this study are as follows: (1) explicit and implicit measures in this study have assessed independent and complementary aspects of forgiveness tendency in older adults. (2) Implicit forgiveness falls behind explicit forgiveness, and true internal forgiveness is difficult and rare in older adults according to data analysis. (3) The trend of explicit forgiveness with age is not obvious, because explicit forgiveness in the middle old age group presents an inflection point. However, implicit forgiveness increases slowly with age. (4) Women excel men in scores obtained with both explicit and implicit measures for forgiveness.

## Data Availability Statement

The raw data supporting the conclusions of this article will be made available by the authors, without undue reservation.

## Ethics Statement

The studies involving human participants were reviewed and approved by the Ethics Review Form for Biomedical Studies at NNU. The patients/participants provided their written informed consent to participate in this study.

## Author Contributions

LT was responsible for the study design, implementation, analysis, interpretation of data, drafting the work and revising it critically for important intellectual content, and reprocesses the data as revising the manuscript. TZ participated in the whole process of writing the manuscript, working hard with the LT on the final revision of the manuscript and responsible for the submission process as well as she was accountable for all aspects. YM undertook a lot of data processing in the process of revising the manuscript. MJ provided suggestions in the preliminary manuscript and especially key ideas and literature in the process of manuscript modification. All authors contributed to manuscript revision, read, and approved the submitted version.

## Conflict of Interest

The authors declare that the research was conducted in the absence of any commercial or financial relationships that could be construed as a potential conflict of interest.

## Publisher’s Note

All claims expressed in this article are solely those of the authors and do not necessarily represent those of their affiliated organizations, or those of the publisher, the editors and the reviewers. Any product that may be evaluated in this article, or claim that may be made by its manufacturer, is not guaranteed or endorsed by the publisher.
